# Editorial: Efficient artificial intelligence in ophthalmic imaging, volume II

**DOI:** 10.3389/fmed.2026.1897768

**Published:** 2026-06-29

**Authors:** Yanda Meng, Meng Wang, Haoyu Chen, Yukun Zhou, Uazman Alam

**Affiliations:** 1 Department of Computer Science, University of Exeter, Exeter, United Kingdom; 2 Department of Cardiovascular and Metabolic Medicine, University of Liverpool, Liverpool, United Kingdom; 3 Liverpool Centre for Cardiovascular Sciences, University of Liverpool and Liverpool University Hospitals NHS Foundation Trust, Liverpool, United Kingdom; 4 Centre for Innovation and Precision Eye Health, National University of Singapore, Singapore, Singapore; 5 Joint Shantou International Eye Center, Shantou University and the Chinese University of Hong Kong, Shantou, China; 6 Department of Ophthalmology & Visual Sciences, The Chinese University of Hong Kong, Hong Kong, Hong Kong SAR, China; 7 Institute of Ophthalmology, University College London, London, United Kingdom

**Keywords:** AI assistance, AI in healthcare, AI in medical diagnosis, medical image, ophthalmology imaging

Few areas of medical Artificial Intelligence (AI) have moved as fast as ophthalmic imaging, and the reasons are not mysterious. The retina is photographed non-invasively, in high resolution, and at a scale few other organs allow, and it carries signatures not only of eye disease but of the vasculature and nervous system behind it. The predictable result has been a long run of papers reporting near-ceiling accuracy. However, the slower, less celebrated work—getting those models to behave on imperfect images, on unfamiliar devices, and inside a working clinic—has lagged behind. This Research Topic was assembled around that gap. The eleven contributions gathered here range from a new transformer architecture to a hospital-wide deployment and, read together, they outline what it now takes to move an ophthalmic algorithm from a leaderboard to a patient.

Diabetic retinopathy (DR) remains the field's proving ground, and three contributions approach it from different distances. Zafar et al. propose a deliberately lightweight two-stage network that first separates healthy from diseased fundus images and then grades severity through transfer learning, reaching 99.06% for detection and 90.75% for five-class grading on APTOS 2019—useful evidence that parameter efficiency and accuracy need not be traded against each other. Ke et al. step back and survey AI across proliferative DR, from diagnosis through surgical planning and anti-Vascular Endothelial Growth Factor optimization, and are refreshingly candid about how little of it has cleared the translation barrier. Umair et al. extend detection to Optical Coherence Tomography (OCT) with OculusNet, identifying choroidal neovascularization, diabetic macular edema, and drusen, and pair the model with saliency maps and a working web interface so that a prediction arrives with both a reason and a route to use.

The Research Topic also expands the field. Gu et al. build a multimodal model for neuromyelitis optica that fuses ultra-widefield fundus photographs with clinical examination data, reaching an Area Under the Curve of 0.9923 for a condition that, until now, had no validated imaging algorithm at all. Al Younis et al. move further from the eye itself, treating retinal OCT thickness metrics as non-invasive markers of heart-failure subtype and adding explainable ML to surface which retinal layers carry the signal. Oculomics of this sort is exactly where the retina-as-window claim gets stress-tested, and the measured framing here, of small cohorts with exploratory conclusions, is appropriate.

A second group confronts the fact that clinical images rarely contain a single tidy finding. Zhang et al. recast multi-label diagnosis as a graph problem in Med-DGTN, combining a dynamic graph transformer with wavelet-based feature fusion to model how diseases co-occur and validate it across both retinal and chest X-ray datasets. Lian et al. adapt a vision transformer with hierarchical attention to grade periocular aging from smartphone photographs, adding region- and patch-level branches that let it focus on subtle detail. Wu et al. turn to the eyelid margin, using a dual-branch U-Net with multi-task attention to segment the palpebral fissure and cornea and then quantify curvature, with agreement against manual annotation strong enough to inform surgical planning. Different problems, one instinct: shape the architecture around the clinical question rather than forcing the question into an off-the-shelf classifier.

Some of the most useful contributions here concern the routine prerequisites. Xia et al. show that a dedicated artifact-removal module—not a heavier classifier—is what lets a real-world ultra-widefield screening system recognize lesions dependably, lifting downstream accuracy by close to 20% on external data. Wan et al. ask the question most AI papers quietly skip: are the measurements even comparable across instruments? Their finding that SD-OCT and SS-OCT macular thickness values agree poorly, and should not be used interchangeably, is a warning for those who train on one scanner and deploy on another. And Pinto et al. supply the study the field needs more of: a before-and-after evaluation of a custom DR tool integrated into general-practitioner workflows across nearly 43,000 screened patients, measuring not a headline accuracy but its effect on referral behavior and reading workload.

Set side by side, the Research Topic makes a case that no single paper makes alone. Strong metrics have become the price of entry rather than the achievement. What separates the work that feels ready from the work that feels promising is attention to everything around the model: the quality of the input, the consistency of the instrument, the legibility of the output, and the workflow it has to survive. A few themes recur across the issue: explainability handled as a feature, multimodal and multi-task formulations becoming the default for messy data, and external or real-world validation treated as expected rather than optional. The unfinished business is just as visible. Cohorts are mostly small and single-region, prospective and regulatory evidence is thin, and the device-consistency problem Wan et al. raise sits uneasily beneath the assumptions of almost every other paper here.

